# Functions and dysfunctions of oligodendrocytes in neurodegenerative diseases

**DOI:** 10.3389/fncel.2022.1083159

**Published:** 2022-12-20

**Authors:** Seungwan Han, Yunho Gim, Eun-Hae Jang, Eun-Mi Hur

**Affiliations:** ^1^Laboratory of Neuroscience, College of Veterinary Medicine and Research Institute for Veterinary Science, Seoul National University, Seoul, South Korea; ^2^BK21 Four Future Veterinary Medicine Leading Education and Research Center, College of Veterinary Medicine, Seoul National University, Seoul, South Korea; ^3^Comparative Medicine Disease Research Center, Seoul National University, Seoul, South Korea; ^4^Interdisciplinary Program in Neuroscience, College of Natural Sciences, Seoul National University, Seoul, South Korea

**Keywords:** oligodendrocyte, neurodegenerative disease, multiple system atrophy, Alzheimer’s disease, Parkinson’s disease

## Abstract

Neurodegenerative diseases (NDDs) are characterized by the progressive loss of selectively vulnerable populations of neurons, which is responsible for the clinical symptoms. Although degeneration of neurons is a prominent feature that undoubtedly contributes to and defines NDD pathology, it is now clear that neuronal cell death is by no means mediated solely by cell-autonomous mechanisms. Oligodendrocytes (OLs), the myelinating cells of the central nervous system (CNS), enable rapid transmission of electrical signals and provide metabolic and trophic support to neurons. Recent evidence suggests that OLs and their progenitor population play a role in the onset and progression of NDDs. In this review, we discuss emerging evidence suggesting a role of OL lineage cells in the pathogenesis of age-related NDDs. We start with multiple system atrophy, an NDD with a well-known oligodendroglial pathology, and then discuss Alzheimer’s disease (AD) and Parkinson’s disease (PD), NDDs which have been thought of as neuronal origins. Understanding the functions and dysfunctions of OLs might lead to the advent of disease-modifying strategies against NDDs.

## 1 Introduction

Due to the lack of electrical activity, it was previously assumed that glial cells functioned as nerve-glue and performed housekeeping functions (Virchow et al., 1860). However, it is now clear that glia are key components of the nervous system, and their roles go far beyond housekeeping. Glia perform vital tasks including regulatory roles in neural circuit formation, synaptic transmission, ion homeostasis, metabolic support, and waste disposal (Allen and Lyons, 2018; Kato and Wake, 2021). Neurodegenerative diseases (NDDs) are characterized by the progressive loss of selectively vulnerable populations of neurons, and clinical symptoms manifested in each NDD depend on neurons that are primarily affected. Accordingly, NDD research focused on cell-autonomous neuronal mechanisms that lead to neuronal dysfunction and death. However, as in many other physiological and pathological processes, increasing evidence suggests that glia play much more active roles in NDDs than originally envisioned and may causally contribute to the onset and progression of NDDs.

Oligodendrocytes (OLs) are highly specialized cells that wrap axons with multiple myelin sheaths, which enable fast and efficient transduction of electrical signals. In the human brain, OLs comprise the most frequent glial cell population comprising 45–75% of glial cells, followed by astrocytes (19–40%) (von Bartheld et al., 2016). OL progenitor cells (OPCs) are present in the white and the gray matter, and they represent the largest population of dividing cells in the adult central nervous system (CNS) (Geha et al., 2010). OPCs retain the potential to proliferate, differentiate, and generate myelin-forming OLs throughout life (Rivers et al., 2008; Gibson et al., 2014). In the adult brain, new OLs are continually produced, and although the efficiency may not be high, newly formed OLs can become stably integrated into mature neural circuits and contribute to the generation of new myelin sheaths (Yeung et al., 2014; Hughes et al., 2018; Franklin et al., 2021). In addition to the formation and repair of myelin, oligodendroglia play a role in immunomodulation (Kirby and Castelo-Branco, 2021) and assure the long-term integrity of axons by providing metabolic and trophic support (Nave, 2010; Narine and Colognato, 2022), all of which are particularly relevant in the context of NDDs.

Multiple sclerosis (MS) is a neurodegenerative, demyelinating, and chronic inflammatory disease of the CNS, typically affecting young adults (Thompson et al., 2018; Tintore et al., 2019; McGinley et al., 2021). The pathological hallmark of MS is focal demyelinating lesions in the white and the gray matter of the brain and the spinal cord, and MS represents a disease in which OLs and myelin are prime targets of pathology (Filippi et al., 2018). Chronic demyelination is thought to trigger a pathogenic cascade of events that cause neurodegeneration: the loss of trophic and metabolic support from OLs, redistribution of ion channels along denuded axons, mitochondrial dysfunction, and enhanced oxidative stress have been proposed as potential mechanistic links leading to axon degeneration and neuronal cell death following myelin loss (Reich et al., 2018; Correale et al., 2019). In recent years, myelin loss and OL dysfunction have been implicated in many other NDDs, not only in the diseases where OL pathology is prominent, such as multiple system atrophy (MSA), but also in NDDs that have been thought of as primarily neuronal origins, such as Alzheimer’s disease (AD) and Parkinson’s disease (PD), two of the most prevalent NDDs. Thus, the relationship among oligodendroglia dysfunction, myelin loss, and neurodegeneration hinted from MS studies might be more general and applied to many other NDDs.

Here we discuss the role of oligodendroglia, which include OPCs and OLs, in NDDs, especially those associated with aging. Increasing evidence suggests that oligodendroglia respond sensitively to NDD pathology, and play an active role in the initiation and progression of NDDs. Deciphering the molecular mechanisms that regulate oligodendroglia function is likely to provide novel insights into developing regenerative therapies that may attenuate, halt, or even reverse the progression of NDDs.

## 2 OL and myelin dysfunction in NDDs

### 2.1 Multiple system atrophy

MSA is a devastating, adult-onset NDD in which multiple neuronal pathways degenerate, causing a multifaceted clinical presentation, including parkinsonism, cerebellar impairment, and autonomic dysfunctions in various combinations (Fanciulli and Wenning, 2015; Poewe et al., 2022). The pathological hallmark of MSA is the presence of glial cytoplasmic inclusions (GCIs), predominantly in OLs (Papp et al., 1989). GCIs are composed of α-synuclein (α-Syn) aggregates, classifying MSA as a synucleinopathy together with PD and dementia with Lewy bodies (LBs) (Malfertheiner et al., 2021). MSA histopathology also includes neuronal and astroglial inclusions, selective neuronal loss and axon degeneration, and myelin pallor accompanying gliosis (Jellinger, 2018). Controversy exists over whether myelin loss is the initial event that leads to neuronal cell death, or whether it is a secondary event as a result of neuronal degeneration, but evidence from postmortem studies and preclinical models supports the view that MSA is a primary oligodendrogliopathy (Fanciulli and Wenning, 2015; Ettle et al., 2016b). Consistent with this notion, restoring oligodendroglial function and enhancing the formation of new myelin have been suggested as a promising approach that may slow or halt disease progression (Ettle et al., 2016a).

Postmortem studies of MSA brains have shown significant correlations between GCI density and the severity of striatonigral degeneration and olivopontocerebellar atrophy (Inoue et al., 1997; Ozawa et al., 2004), as well as the correlation between GCI pathology and disease duration (Ozawa et al., 2004). In MSA, neuronal pathology in the form of α-Syn-immunoreactive inclusions has also been reported and might be widespread (Cykowski et al., 2015). However, the topography of neuronal inclusions is poorly defined and their distribution and frequency show little correlation with neuronal cell death or the clinical symptoms of MSA.

Capturing the earliest changes, especially those that occur prior to the manifestation of MSA pathology might provide critical insights into the primary trigger of the disease. Although much can be learned from autopsy studies, postmortem data are not longitudinal in nature. To understand how the disease progresses and to investigate the cause-effect relationship between GCI and neurodegeneration, several rodent models of MSA designed to express human α-Syn in OLs have been generated. They develop GCI-like inclusions within OLs and exhibit neurodegeneration, myelin abnormalities, and/or motor deficits (Kahle et al., 2002; Shults et al., 2005; Yazawa et al., 2005). Although these transgenic lines force OLs to overexpress α-Syn, studies from these models suggest that the formation of GCI is sufficient to drive neurological dysfunction.

#### 2.1.1 Progression of α-Syn pathology

α-Syn is produced primarily in neurons, and oligodendroglia do not normally express the protein (Solano et al., 2000; Miller et al., 2005). Although controversial, a few studies reported the presence of *SNCA* mRNA in OLs (Asi et al., 2014; Djelloul et al., 2015), suggesting the possibility that endogenous α-Syn in OLs might provide a source for GCIs. The mechanisms responsible for the accumulation of α-Syn in GCIs within OLs are unclear, but two hypotheses have been proposed. One possibility is that OLs pathologically increase the expression of α-Syn in MSA brains. However, postmortem studies of MSA brains failed to elucidate the increase of *SNCA* in OLs (Ozawa et al., 2001; Honglian et al., 2008; Asi et al., 2014). Another possibility is that OLs take up α-Syn secreted from neurons. Several studies have shown that OLs can internalize diverse forms of α-Syn and that neuronal α-Syn can be transferred to OLs (Kisos et al., 2012; Konno et al., 2012; Reyes et al., 2014). It is still enigmatic how α-Syn pathology propagates and selectively accumulates among oligodendroglia in MSA brains, but in the context of MSA, OLs might become more vulnerable to the invasion of α-Syn, and the mechanisms responsible for clearing pathogenic α-Syn might operate less effectively compared to neurons.

Tubulin polymerization promoting protein (TPPP)/p25α, an OL-specific phosphoprotein, is a component of GCI (Kovács et al., 2004) and has been suggested to contribute to the formation of OL-specific α-Syn strain (Ferreira et al., 2021). TPPP/p25α is known to play a role in organizing cellular microtubule network and promoting OL differentiation (Lehotzky et al., 2010; Fu et al., 2019). In normal brains, TPPP/p25α colocalizes with myelin basic protein (MBP), but in MSA brains, TPPP/p25α is found in the cell body (Song et al., 2007). Translocation of TPPP/p25α precedes GCI formation (Song et al., 2007), and this relocalization is thought to promote α-Syn aggregation by stabilizing unstructured α-Syn (Lindersson et al., 2005; Szunyogh et al., 2015). TPPP/p25α has also been suggested to counteract α-Syn degradation by inhibiting the maturation of autophagosome and its fusion with the lysosome (Ejlerskov et al., 2013; Lehotzky et al., 2021). TPPP/p25α bears a KFERQ-like motif and could be recognized and cleared via chaperone-mediated autophagy (Mavroeidi et al., 2022). These studies suggest that enhancing the autophagy-lysosome pathway might prevent GCI formation by facilitating the clearance of α-Syn and TPPP/p25α in OLs.

**FIGURE 1:**
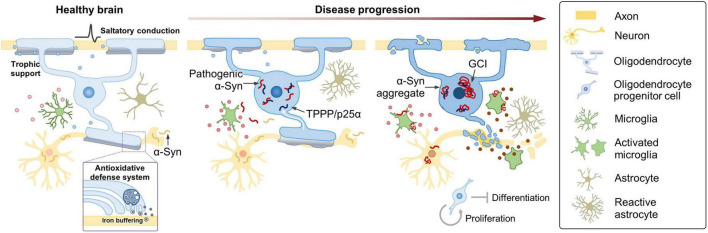
Oligodendroglial pathology and possible mechanisms contributing to MSA. Under healthy conditions, OLs enable saltatory conduction of electrical signals, provide metabolic and trophic support, and play a role in providing an antioxidant defense system to neurons. In MSA, TPPP/p25α relocates from the myelin sheath to the cell body, prior to α-Syn aggregation. Redistribution of TPPP/p25α causes cellular enlargement, and α-Syn starts to accumulate in the enlarged cytoplasm. Metabolic and trophic support of OLs to neurons is compromised, iron overload increases, and oxidative stress becomes aggravated. At later stages, α-Syn aggregates form GCIs in OLs. α-Syn is transformed into a toxic form in the intracellular environment of OL, where α-Syn seems to acquire distinct characteristics of MSA. MSA-type α-Syn might be transferred to neurons, and pathogenic α-Syn may induce neurodegeneration by directly interfering with essential neuronal functions or indirectly via aberrant activation of micro- and astrogliosis. As the disease progresses, microglia become destructive by releasing neurotoxic inflammatory mediators, such as cytokines, chemokines, and reactive oxygen and nitrogen species. The changing state of microglia, reflected in part, by their secretory profile, might play a crucial role in the progression of MSA. Enhanced proliferation of OPCs occurs, perhaps in an attempt to compensate for the myelin loss, but under the burden of α-Syn, immature oligodendroglia fail to differentiate and undergo cell death.

Accumulation of α-Syn in OLs has been shown to cause demyelination, iron overload, and exacerbate autophagy impairment (Poewe et al., 2022). Interestingly, in contrast to the severe neuronal loss, previous studies have reported that MSA brains show only a modest reduction in the number of OLs (Salvesen et al., 2015; Nykjær et al., 2017), suggesting that cellular dysfunction of OLs, and not necessarily cell death, induced by GCIs may be sufficient to cause neurodegeneration. These results were in disagreement with earlier studies documenting apoptotic cell death of OLs in MSA brains (Probst-Cousin et al., 1998; Kragh et al., 2009, 2013). Further studies are needed to thoroughly examine if the accumulation of α-Syn causes oligodendroglial death in MSA brains and if the death of GCI-positive OLs precedes and mediates neurodegeneration. Accumulation of α-Syn has also been detected in PDGFRα^+^ OPCs and BCAS1^+^ immature oligodendroglia in the brains of MSA patients and MSA transgenic mice overexpressing human wild-type α-Syn under the control of MBP promoter (May et al., 2014; Kaji et al., 2020). In MSA brains, the number of OPCs was increased (Ahmed et al., 2013; May et al., 2014), reminiscent of the increase in OPC number in a variety of other CNS damages (Keirstead et al., 1998; Schönrock et al., 1998; Hampton et al., 2004; Magnus et al., 2008). Enhanced proliferation of OPCs might occur in an attempt to compensate for the myelin loss (Franklin et al., 2021). However, under the burden of α-Syn, immature oligodendroglia cannot differentiate into mature OLs (Ettle et al., 2016a) and undergo cell death (May et al., 2014; Kaji et al., 2020), and thus fail to replace the defective OLs. Defective oligodendroglia will not be able to provide sufficient trophic and metabolic support to neurons (Jha and Morrison, 2020), and pathogenic α-Syn may induce neurodegeneration by directly disrupting essential cellular functions in neurons (Wong and Krainc, 2017) and indirectly via the aberrant activation of micro- and astrogliosis (Harms et al., 2021; Figure 1).

Both intraneuronal LBs, the pathological hallmark of PD, and GCIs are composed of α-Syn aggregates. However, MSA and PD show prominent differences not only in the clinical manifestations and the rate of disease progression, but also in the brain regions and cell types vulnerable to the deposition of α-Syn. It has been suggested that pathological α-Syn in GCIs and LBs are conformationally and biologically distinct (Peng et al., 2018; Lau et al., 2020), with GCI-derived α-Syn aggregates being much more potent inducers of pathology than LB-derived aggregates (Peng et al., 2018). Peng et al. (2018) provided evidence that the different intracellular environments of OLs and neurons convert α-Syn seeds to different strains. Neuronal cytoplasmic inclusions (NCIs) are observed in MSA brains, albeit less frequently. The ultrastructure of NCIs resembles that of GCIs, rather than that of LBs from PD brains (Takeda et al., 1997), and filaments in GCIs and NCIs are morphologically indistinguishable (Murayama et al., 1992). In MSA, neurons are the likely source for the initial α-Syn (see above), but α-Syn is somehow transformed into a toxic form in the intracellular environment of OLs, where α-Syn seems to acquire distinct characteristics of MSA. To better understand MSA pathology, it will be of great importance to reveal the molecular determinants and cellular processes in the intracellular milieu of OLs that confer toxicity.

### 2.2 Alzheimer’s disease

AD is the most common age-related NDD (Alzheimer’s Association, 2019; Hou et al., 2019). AD presents cognitive and executive dysfunctions and is biologically defined by the presence of amyloid beta (Aβ) plaques and neurofibrillary tangles containing tau (Long and Holtzman, 2019; Knopman et al., 2021). Although AD is considered as a gray matter disease, postmortem AD brains clearly show white matter damages (Scheltens et al., 1995; Gouw et al., 2008; Nasrabady et al., 2018), and a number of studies have shown that the extent of myelin loss correlates with Aβ burden (Grimmer et al., 2012; Brickman et al., 2015a; Dean et al., 2017). Recently, single-cell transcriptomic analyses of individuals with AD pathology have revealed that oligodendroglia show pathology-responsive transcriptional signatures, in addition to neurons and microglia, the cell types which have been the primary focus in the AD research field (Grubman et al., 2019; Mathys et al., 2019; Chen et al., 2020). Of note, myelination-related genes were among the top differentially expressed genes perturbed in most major cell types in AD brains, suggesting that myelination has a key role in AD pathophysiology (Mathys et al., 2019). Postmortem brain samples from AD patients with the common variant of *TREM2* also showed significant changes in OLs: genes involved in differentiation were downregulated, whereas those related to lipid metabolism and oxidative stress were elevated, indicative of impaired myelination and metabolic adaptation to neuronal degeneration (Zhou et al., 2020).

**FIGURE 2:**
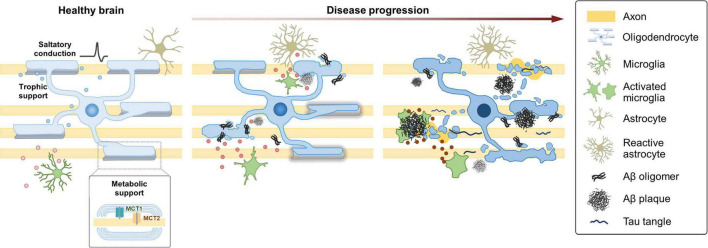
Dynamic changes of oligodendroglia during the progression of AD. Under healthy conditions, OLs enable saltatory conduction of electrical signals and provide metabolic and trophic support to neurons. In the AD brain, OLs are vulnerable to neurodegenerative conditions and acquire a distinct cellular state in response to the changing microenvironment. Under mild Aβ exposure, OLs alter gene expression and start to exhibit myelin defects. As the disease progresses, defects in OL function and myelin loss become more evident in correlation with the increased Aβ burden. Perturbation of myelin-axon coupling affects signal propagation as well as metabolic and trophic support. At later stages with higher Aβ burden, a specific subgroup of OLs express *C4b* and *Serpina3n*. Some aspects of the late-stage OLs might be shared across several NDDs. As AD progresses, microglia also become destructive by releasing neurotoxic inflammatory mediators, such as cytokines, chemokines, and reactive oxygen and nitrogen species. The changing state of microglia, reflected in part, by their secretory profile, might play a crucial role in the progression of AD.

Animal models of AD have greatly enhanced the understanding of the molecular and cellular mechanisms of the disease. Despite that existing models have focused largely on neurons with a particular emphasis on proteinopathy, several models exhibit defects or changes in oligodendroglia (Desai et al., 2009; Gu et al., 2018; Chacon-De-La-Rocha et al., 2020). A single-cell transcriptomic study using 5XFAD mice—a murine model of amyloidosis carrying five familial AD mutations—identified a novel OL population responding to Aβ accumulation, defined by the expression of the complement component C4b and the serine protease inhibitor Serpina3n (Zhou et al., 2020). Other studies revealed the presence of distinct but shared OL populations, termed “disease-associated OLs,” detected across many different pathological models, including mouse models of not only tauopathy and amyloidosis but also MS (Kenigsbuch et al., 2022; Pandey et al., 2022). Despite that the molecular signatures of OLs in human AD seemed to be largely distinct from those observed in mouse models (Zhou et al., 2020; Pandey et al., 2022), it is clear that OLs are vulnerable to neurodegenerative conditions and acquire a distinct cellular state in response to the changing microenvironment. A recent spatial transcriptomics study using brains from *APP^NL– G– F^* knock in mice (carrying Swedish KM670/671NL, Arctic E22G, and Beyreuther/Iberian I716F mutations) has demonstrated early alterations in a gene expression network enriched for myelin and OL genes (Chen et al., 2020). The gene set termed “OLIG module” mainly expressed by OLs was highly expressed under mild Aβ exposure but became downregulated in microenvironments with dense Aβ accumulation (Chen et al., 2020). Together with the postmortem analyses of human AD, data from animal models provide strong support for the notion that OLs dynamically respond to AD pathology (Figure 2).

In AD patients, white matter abnormalities can be detected at least a decade before clinical manifestations (Mortamais et al., 2013; Lee et al., 2016; Araque Caballero et al., 2018), and in AD mouse models, myelin disruption can be observed prior to the appearance of plaques and tangles (Desai et al., 2009). Moreover, white matter abnormalities are associated with the risk and the onset of AD (Tosto et al., 2014; Brickman et al., 2015b; Lee et al., 2016). These lines of evidence support the notion that OLs not only respond to pathology but also causally contribute to AD. Further studies are needed to examine if OL-specific gene expression can be detected in various models of AD, especially at early stages, as well as in sporadic AD and familial AD patients with other mutations to ensure that the transcriptional changes in OL lineage cells actually reflect a significant process during AD progression. More importantly, to thoroughly examine what roles oligodendroglia play in the neurodegenerative process and to investigate whether the changes in oligodendroglia are causative, it will be imperative to perform longitudinal studies in new models where initial changes take place in OPCs and/or OLs.

#### 2.2.1 Possibility of targeting oligodendroglia for the treatment of AD

Given that myelin defect is highly associated with cognitive dysfunction, promoting myelination might provide a way to ameliorate clinical symptoms of AD. Recent studies showed that enhancing myelin renewal, by deletion of the muscarinic M1 receptor in oligodendroglia or administration of clemastine, rescued deficits in cognition and hippocampal physiology in APP/PS1 mouse—an AD model overexpressing human amyloid precursor protein (APP) and presenilin-1 (PS1) with mutations associated with early onset familial AD (Chen et al., 2021; Xie et al., 2021). To establish myelin loss as a major contributor to cognitive impairment in the context of AD, temporal dynamics of demyelination should be examined in other AD models, including those modeling tau pathology (Yoshiyama et al., 2007) and second-generation mouse models that have been generated to overcome the intrinsic drawbacks of the overexpression paradigm (Sasaguri et al., 2017).

Zhang et al. (2019) reported the presence of OPCs exhibiting a senescence-like phenotype characterized by the expression of p21/CDKN1A and p16/INK4/CDKN2A proteins in the Aβ plaque environments of brains of AD patients and the APP/PS1 mice. They also showed that the administration of senolytic drugs removed senescent OPCs from the brain, prevented plaque formation, and improved cognition in APP/PS1 mice. The exact mechanisms by which senescent OPCs contribute to the pathogenesis of AD remain to be investigated, but it is plausible to suggest that secretory profiles of the senescent OPCs play a role in triggering or enhancing local inflammation, demyelination, and/or neuronal dysfunction. In addition to serving as a reservoir for remyelination, OPCs have emerged as active participants in multiple aspects of brain function. OPCs receive synaptic information from neurons (Lin and Bergles, 2004), associate closely with the blood-brain barrier (BBB) (Seo et al., 2013), and play a role in immunomodulation by monitoring the environment and releasing various factors (Falcao et al., 2018; Kirby et al., 2019). Given the involvement of synaptic loss (Subramanian and Tremblay, 2021), BBB breakdown (Knox et al., 2022), and chronic inflammation (Stephenson et al., 2018) in the progression of AD as well as other NDDs, understanding the roles of OPCs and how they become dysregulated is likely to lay the groundwork for a new era of NDDs.

### 2.3 Parkinson’s disease

PD is the most common neurodegenerative movement disorder (Feigin et al., 2019). The loss of dopaminergic neurons in the substantia nigra (SN) pars compacta and the deposition of α-Syn in neurons are neuropathological hallmarks of PD (Poewe et al., 2017). For PD, highly efficacious therapies to increase striatal dopamine levels are available, such as pharmacological dopamine substitution and deep brain stimulation (Poewe et al., 2017), providing an unrivaled example of an NDD that can be effectively managed. However, none of these treatments slow down disease progression, and there remains a critical unmet need for disease-modifying therapies that can delay, prevent, or reverse disease progression.

A recent study integrating genome-wide association studies with single-cell transcriptomic data from the mouse nervous system revealed that PD is genetically associated not only with cholinergic and monoaminergic neurons, as expected, but also, unexpectedly, with OLs (Bryois et al., 2020). Interestingly, genes upregulated in PD were specifically expressed in OLs, while the downregulated genes were enriched in dopaminergic neurons. Moreover, using postmortem brain transcriptomic data, Bryois et al. (2020) confirmed that those upregulated genes in OLs were elevated across all Braak stages, even in the earliest stages of the disease. These results suggest that changes in OLs precede the emergence of pathological traits, giving new insights into the causes of PD. Consistent with these results, Agarwal et al. (2020) performed single-nuclei transcriptomics of the human SN and found that the genetic risk of PD was associated with OLs and OPCs, in addition to dopaminergic neurons. The fraction of PD genetic risk associated with OLs was distinct from that fraction associated with dopaminergic neurons (Agarwal et al., 2020), suggestive of distinct PD-associated cell etiologies. They also observed that the expression of *LRRK2* gene, which encodes leucine-rich repeat kinase 2 and which is associated with both familial and sporadic PD (Hur et al., 2019; Hur and Lee, 2021), was significantly higher in OPCs than in any other cell types in the SN (Agarwal et al., 2020). The physiological and pathological relevance of the manifestation of PD genetic risk through OLs and OPCs remains to be investigated, but it might reflect the role of oligodendroglia in controlling the local environment and regional vulnerability of the SN.

The myelination status or the degree of myelination has been suggested as a key factor determining neuronal vulnerability to Lewy pathology in PD (Braak et al., 2006; Orimo et al., 2011), along with other features, such as long highly branched axons, autonomous activity, calcium-dependent pacemaking, and high levels of oxidative stress (Braak et al., 2004; Chan et al., 2009). In postmortem studies, the accumulation of α-Syn was more evident in unmyelinated or poorly myelinated axons (Braak et al., 1999; Volpicelli-Daley et al., 2014). It is unclear if axonal hypomyelination aggravates α-Syn pathology or if the axonal accumulation of α-Syn affects membrane status or myelination, but Lewy neurites might reflect pathological changes in the structural or functional interaction between the axon and surrounding myelin. In fact, white matter abnormalities have often been reported in PD patients (Karagulle Kendi et al., 2008; Zhan et al., 2012; Melzer et al., 2013; Duncan et al., 2016). The pathogenesis is poorly understood, but white matter damage is detected in newly diagnosed PD patients and has been reported to occur before gray matter atrophy can be observed (Agosta et al., 2014; Duncan et al., 2016). A recent study taking a whole-brain connectomics approach also suggested a significantly decreased myelin content in PD patients compared with control subjects, especially along the connections emerging from the SN (Boshkovski et al., 2022). Despite the increasing evidence suggesting the possible involvement of oligodendroglia in PD, OLs have received far less attention in PD compared to MSA and AD. Further studies are needed to investigate how OL lineage cells are affected in PD and to explore if specific molecules or functional pathways in oligodendroglia can be targeted for therapeutic interventions.

## 3 Possible impact of defective myelin-axon coupling on neurodegeneration

Demyelination and dysfunction of oligodendroglia have been implicated in a number of NDDs, but it is difficult to ascertain whether defective oligodendroglia play a causal, or at least a primary role, in early disease stages. Oligodendroglia have long been suggested to provide trophic and metabolic support for neuronal survival. Mutant mice lacking proteolipid protein, a major myelin membrane protein, show late-onset axonal degeneration (Klugmann et al., 1997; Griffiths et al., 1998), and mice lacking 2’, 3’-cyclic nucleotide 3’-phosphodiesterase, another myelin protein, exhibit abnormal axonal swelling and degeneration in the absence of major myelin alterations (Lappe-Siefke et al., 2003). These studies suggest that support from OLs is essential for long-term axonal survival (Micu et al., 2018), but also raise the question regarding the underlying mechanisms of OL-axon interactions. One way that OLs provide support to axons is via monocarboxylate transporters (MCTs), localized in myelin (MCT1) and on the axolemma (MCT2), which function to fuel lactate (Lee et al., 2012). It has been shown that disruption of MCT1 causes axonal damage and neuronal loss, and that MCT1 is reduced in patients with amyotrophic lateral sclerosis (Lee et al., 2012). The contribution of oligodendroglial MCTs to other NDDs requires further study. To determine whether oligodendroglia play a primary role in early stages of NDDs, it will be imperative to reveal the actual factors provided by OLs to nearby neurons and the mechanisms of interaction.

Postmortem studies have shown increased iron content in specific regions of MSA, PD, and AD brains (Raven et al., 2013; Carocci et al., 2018; Mochizuki et al., 2020). Although further study is needed to determine whether iron dyshomeostasis has a causal role and to investigate how much it contributes to the aggravation of neurodegeneration, there is evidence that iron dyshomeostasis is present at early stages in many NDDs (Ndayisaba et al., 2019). In the adult brain, OPCs and mature OLs are the cells with the highest iron levels (Connor and Menzies, 1996; Reinert et al., 2019). Oligodendroglia require iron as a cofactor for several enzymes that regulate the proliferation and differentiation of OPCs, as well as the production and maintenance of myelin (Todorich et al., 2009). The disturbance of iron homeostasis leads to the generation of free radicals and oxidative stress. Ferroptosis is a recently discovered type of cell death caused by the iron-dependent accumulation of lethal amounts of lipid-based reactive oxygen species (Dixon et al., 2012; Stockwell et al., 2017). Given the high intracellular iron concentration in oligodendroglia and low levels of antioxidative agents (such as glutathione and mitochondrial manganese superoxide dismutase) coupled with their high metabolic rate, it is tempting to speculate that in NDDs, oligodendroglia are extremely vulnerable to iron-induced oxidative damage and may undergo ferroptosis. Neurons are also sensitive to oxidative stress, and a recent study suggested that OLs play a role in protecting neighboring neurons from iron-mediated cytotoxicity via secretion of ferritin heavy chain (Mukherjee et al., 2020). It will be interesting to explore if ferroptosis in oligodendroglia and neurons plays a role in the pathogenesis of NDDs.

## 4 Uncovering the heterogeneity of oligodendroglia and the impact of aging

Recent studies have revealed that oligodendroglia comprise a heterogeneous population (Zeisel et al., 2015; Marques et al., 2016). OPCs and OLs might become morphologically and/or functionally diversified not only during development (Marques et al., 2018) but also during the course of diseases (Zhou et al., 2020). It remains to be determined if OPCs with certain genetic and molecular signatures have a higher potential to differentiate and myelinate. In the case of MS, it has been suggested that disease pathology results from the loss of a certain subclass of mature OLs rather than an overall failure of OPC differentiation, and thus, a general strategy to enhance OPC differentiation might be insufficient to enhance remyelination (Jakel et al., 2019). Therefore, elucidating heterogeneity and functional differences of the subclasses of oligodendroglia will be of great importance, and strategies to restore the ones that contribute to remyelination should be key. Analyses of AD patient brains and AD mouse models have revealed the presence of a specific population of OLs (Zhou et al., 2020; Kenigsbuch et al., 2022) exhibiting distinct gene expression profiles or phenotypes associated with pathology and/or neurodegeneration. Zhou et al. (2020) suggested that OL signatures associated with AD seemed to be different from those associated with MS (Jakel et al., 2019) or senescence (Zhang et al., 2019). By contrast, Kenigsbuch et al. (2022) suggested the presence of an OL state associated with diverse diseases, which could be found in multiple CNS pathologies, regardless of the nature of the etiology. Future studies are needed to examine whether the disease-responsive signature of OL lineage cells reported in AD models are also found in the context of other NDDs, and investigate to what extent the signatures are etiology-specific and how much they are conserved. Identification of the oligodendroglia population with the responsive vs. driving nature of the disease will also improve the understanding of the molecular and cellular pathways underlying the onset and progression of NDDs.

Advancing age is the single most prominent risk factor for NDDs. Although studies of OPC proliferation and differentiation in the developing nervous system have suggested a number of pathways that can be targeted to enhance remyelination in the mature nervous system (Fancy et al., 2011; Ishino et al., 2022), increasing evidence suggests that young and old OPCs are not identical (Segel et al., 2019; de la Fuente et al., 2020). In fact, a substantial loss of myelinated fibers occurs with aging (Marner et al., 2003), and remyelination is thought to be one of the major processes in the brain affected by aging (Sim et al., 2002; Neumann et al., 2019). Decreased remyelination efficiency has been attributed to impaired infiltration of aged OPCs to the lesion area (Sim et al., 2002), reduced responsiveness of aged OPCs to pro-differentiation factors, and their slower inherent capacity for differentiation compared to young OPCs (Neumann et al., 2019). Transcriptomic analysis of OPCs isolated from young and old rats suggested that aged OPCs acquire a variety of hallmarks of aging. Nonetheless, OPCs persist throughout the lifespan (Young et al., 2013), and rodent studies show that aged OPCs can be rejuvenated and manipulated to undergo enhanced differentiation (Neumann et al., 2019; Wang et al., 2020; Iram et al., 2022). In addition to their contribution to remyelination, OPCs are thought to play much more diverse roles including synaptic, vascular, and immunomodulatory functions (Akay et al., 2021). Therefore, OPCs remain attractive targets with immense translational potential to overcome the effects of aging. A recent study suggested that aging was also associated with the induction of distinct subpopulations of mature OLs, one characterized by a high expression of Serpina3n and C4b and a smaller subpopulation characterized by the expression of genes related to an interferon response (Kaya et al., 2022). Much remains to be elucidated regarding how aging alters oligodendroglial lineage cells and how the changes in their cell state contribute to age-related myelin degeneration and insufficient myelin renewal. Developing methods to monitor and manipulate aged and young oligodendroglia *in vitro* and *in vivo*, technologies for *in vivo* imaging, and devising analytic tools to investigate OL physiology and function hold great potential to catalyze exciting studies deciphering functions and dysfunctions of oligodendroglia and their significance in the aging brain and NDD progression.

## 5 Concluding remarks

Despite the increased understanding of genetic factors and molecular mechanisms contributing to NDDs, none of the existing treatments alter the natural course of NDDs, and there has been limited success in translating the knowledge gained from preclinical studies into targeted therapies. NDD research has been neurocentric for decades, but the complexity and heterogeneity of NDDs suggest that NDDs result from the activation of more than one pathogenic pathway, perhaps involving multiple cell types as well as their interplay. Indeed, studies over the last two decades have revealed new pathways and cell types associated with NDDs, and we are beginning to appreciate that the cell types associated with the genetic risk for a particular NDD are not necessarily those cell types most directly affected by the defining pathology or those directly associated with the clinical symptoms. Here we have discussed recent findings suggesting an unexpected role of oligodendroglia, perhaps the cells that received far less attention compared to not only neurons but also other glial cells. Emerging evidence supports the view that OL lineage cells might be one of the most vulnerable cell types responding to the changing microenvironment in the brain during the course of NDDs. Moreover, OPCs and mature OLs might play a modifying role in the onset and progression of NDDs. Given that OPCs exist throughout life and retain the potential to generate new myelin, these cells may serve as reservoirs for regenerative therapy to combat NDDs. Understanding how oligodendroglia are affected in NDDs and how they can be controlled should lead to an in-depth understanding of NDDs and might help develop effective diagnostic and therapeutic approaches.

## Author contributions

SH, YG, and E-MH wrote the first draft of the manuscript. E-HJ conceptualized and prepared the figures. All authors performed a literature search, contributed to revising the article, and approved the submitted version.
